# Robust squeezed light against mode mismatch using a self imaging optical parametric oscillator

**DOI:** 10.1038/s41598-021-98328-7

**Published:** 2021-09-23

**Authors:** Chan Roh, Geunhee Gwak, Young-Sik Ra

**Affiliations:** Department of Physics, Korea Advanced Institute of Science and Technology, Daejeon, 34141 Korea

**Keywords:** Quantum information, Quantum metrology, Quantum optics

## Abstract

We present squeezed light that is robust against spatial mode mismatch (beam displacement, tilt, and beam-size difference), which is generated from a self-imaging optical parametric oscillator below the threshold. We investigate the quantum properties of the generated light when the oscillator is detuned from the ideal self-imaging condition for stable operation. We find that the generated light is more robust to mode mismatch than single-mode squeezed light having the same squeezing level, and it even outperforms the single-mode infinitely squeezed light as the strength of mode mismatch increases.

## Introduction

Squeezed light is a versatile quantum resource for quantum information technologies^1–7^. In particular, in quantum metrology, it reduces noises in measurement below the standard quantum limit^8–10^; a notable application is gravitational wave detectors^11,12^. The quality of squeezed light is typically measured by the squeezing level (the degree of noise reduction compared with the vacuum noise), which is reported up to 15 dB by using an optical parametric oscillator (OPO)^13^.

To exploit the full potential of squeezed light in quantum technologies, however, a precise mode matching with subsequent operations is required^14–16^. Mismatch of modes results in a loss of original properties of light, e.g. degradation of the squeezing level^15^. For classical light, mode mismatch is not a critical issue because the loss of light can be simply compensated by means of increasing the optical power. However, for squeezed light, there is a limit on compensation by increasing the initial squeezing level, e.g., if the mode matching efficiency is less than 50%, there is no way to obtain more than 3 dB squeezing. Moreover, if mode mismatch varies dynamically or even fluctuates stochastically, its correction is a very challenging problem^14,17^.

A way of circumventing this problem is to prepare squeezed light in a few additional modes, so that, in case of mode mismatch, squeezed light from the additional modes is coupled into a target mode^18,19^. Recently, such an idea is demonstrated by employing an additional OPO operating in a Hermite–Gaussian ${\hbox{HG}}_{{01}}$ mode for a vertical mode mismatch^16^. However, to be robust against more general and stronger mode mismatch, it is required to build many phase-locked OPOs, each operating in a different ${\hbox{HG}}_{{mn}}$ mode^20^, which is technically demanding.

Here we show that a single OPO in a self-imaging configuration can generate squeezed light that is robust against various cases of mode mismatch. The self-imaging OPO is based on a fully degenerate optical cavity in spatial modes, called a self-imaging cavity^21,22^, hence it can support spatially multimode quantum states generated by a parametric down-conversion process^23–25^. However, the ideal self-imaging condition leads to cavity instability, and thus, a small detuning is necessary for stable operation^22^. In this work, we characterize the quantum properties of the light from a self-imaging OPO with a small detuning (parametrized by the Gouy phase shift) and an intracavity loss, and we find that squeezed vacua at multiple ${\hbox{HG}}_{{mn}}$ modes are simultaneously generated without much degradation from the ideal self-imaging condition. We then investigate the injection of the generated multimode light into a target mode in the presence of mode mismatch (beam displacement, tilt, and beam-size difference both for horizontal and vertical directions) and optical losses. We find that the multimode squeezed light is more robust to mode mismatch than single-mode squeezed light with the same squeezing level, and at sufficiently large mode mismatch, it even outperforms the single-mode infinitely squeezed light. Such robustness to mode mismatch can be achieved in a detuning range of a self-imaging OPO within reach of the experimental control.

## Quantum properties of multimode squeezed light

### Spatial properties of parametric down conversion

To generate squeezed light, we consider a degenerate type-I parametric down conversion in a $\chi ^{(2)}$nonlinear crystal in the low gain regime^26^. The interaction Hamiltonian can be described as

1 $$\begin{aligned} {\hat{H}} = -{i\hbar{g} \over 2} \int d^{2}{\vec{q}}_{s} d^{2}{\vec{q}}_{i}\, {\tilde{K}}({\vec{q}}_{s},{\vec{q}}_{i}) \, {\hat{a}}^{\dagger }({\vec{q}}_{s}) {\hat{a}}^{\dagger }({\vec{q}}_{i})+h.c., \end{aligned}$$

where ${\vec{q}}_{s(i)}$ is the transverse component of the wave vector for the signal (idler) field, ${\hat{a}}^{\dagger }({\vec{q}})$ is the creation operator at ${\vec{q}}$ and $t=0$, and *g* is the gain parameter proportional to the crystal nonlinearity $\chi ^{(2)}$, the length of the nonlinear crystal $l_{c}$, and the maximum pump amplitude $A_{p}$. The kernel $K({\vec{q}}_{s},{\vec{q}}_{i})$ determines the spatial properties of the generated light, which depends on the pump beam distribution and the phase matching condition. For a monochromatic Gaussian pump beam focused at the center of the crystal, the kernel is given by^23, 27^

2 $$\begin{aligned} {\tilde{K}}({\vec{q}}_{s},{\vec{q}}_{i})&= \exp \left( {-{w_{p}^{2} \over 4}\vert {\vec{q}}_{s}+{\vec{q}}_{i} \vert ^{2}} \right) {\text{sinc}}\left( {l_{c} \over 4k_{p}} \left| {\vec{q}}_{s} - {\vec{q}}_{i} \right| ^{2} \right) , \end{aligned}$$

where $k_{p}$ and $w_{p}$ are the wavenumber and the waist of the pump beam, respectively. The Gaussian function originates from the pump distribution (making the anticorrelation between ${\vec{q}}_{s}$ and ${\vec{q}}_{i}$), and the sinc function is by the phase matching condition (making the correlation between ${\vec{q}}_{s}$ and ${\vec{q}}_{i}$).

The Hamiltonian ${\hat{H}}$ in Eq. (1) can be described by the transverse position operator ${\hat{a}}^\dagger ({\vec{x}}) (= {1 \over 2\pi } \int d^{2}{\vec{q}}\, e^{{-i{\vec{q}}} \cdot {\vec{x}}}\,{\hat{a}}^{\dagger }({\vec{q}}))$ as well, using the inverse Fourier transform,

3 $$\begin{aligned} {\hat{H}} = -{i\hbar{g} \over 2} \int d^{2}{\vec{x}}_{s} d^{2}{\vec{x}}_{i}\, K({\vec{x}}_{s},{\vec{x}}_{i}) \, {\hat{a}}^{\dagger }({\vec{x}}_{s}) {\hat{a}}^{\dagger }({\vec{x}}_{i})+h.c. \end{aligned}$$

The associated kernel $K({\vec{x}}_{s},{\vec{x}}_{i})$ is decomposed with ${\hbox{HG}}_{{mn}}$ functions ($\psi ^H_{mn}(x,y)$) by approximating the sinc function in ${\tilde{K}}({\vec{q}}_{s},{\vec{q}}_{i})$ to the Gaussian function (${\mathrm{sinc}}(x^{2}) \approx \exp (-\alpha x^{2})$)^28,29^,

4 $$\begin{aligned} K({\vec{x}}_{s},{\vec{x}}_{i}) = \sum _{m,n=0}^{\infty }\mu ^{m+n}\, \psi ^H_{mn}({\vec{x}}_{s}) \,\psi ^H_{mn}({\vec{x}}_{i}), \end{aligned}$$

where

5 $$\begin{aligned} \psi ^H_{mn}(x,y)&= { \exp \left( -{{ x^{2}+ y^{2}} \over w_{H}^{2} }\right) H_m\left( {\sqrt{2} x \over w_{H} }\right) H_n\left( { \sqrt{2} y \over w_{H} }\right) \over {w_{H} \sqrt{2^{m+n-1} \pi m!n!}} } \nonumber \\ \mu&= {1 - \sqrt{\xi } \over 1+\sqrt{\xi }} \nonumber \\ \xi&= \alpha l_{c}/2z_{p} \nonumber \\ w_{H}&= \sqrt{2} \xi ^{1/4} w_{p}. \end{aligned}$$

The pump beam property has been expressed in terms of the Rayleigh range $z_{p}= k_{p} w_{p}^{2}/2$, and $H_n(x)$ is the *n*-th order Hermite polynomial. The key parameters determining the kernel are a modified (by $\alpha$) focusing parameter of the pump $\xi$ and the waist size $w_{H}$. $\xi$ determines the eigenvalues $\mu ^{m+n}$, where $\left| {\mu }\right| ^{m+n} < 1$, and $w_{H}$ determines the width of HG modes $\psi ^H_{mn}(x,y)$. The Schmidt number *M*, quantifying the average number of modes^30^, can be also expressed as a function of the focusing parameter $\xi$,

6 $$\begin{aligned} M = {1 \over 4} \left( \sqrt{\xi }+\sqrt{1/\xi }\right) ^{2}. \end{aligned}$$

*M* has the minimum value of one at $\xi =1$, and *M* increases as $\xi$ deviates from one.

With this kernel decomposition, the Hamiltonian in Eq. (3) can be expressed in a decoupled form

7 $$\begin{aligned} {\hat{H}} = -{i\hbar g \over 2} \sum _{mn} \mu ^{m+n} \left( {\hat{A}}^{H}_{mn}\right) ^{\dagger }\left( {\hat{A}}^{H}_{mn}\right) ^{\dagger } + h.c., \end{aligned}$$

where

8 $$\begin{aligned} \left( {\hat{A}}^{H}_{mn}\right) ^{\dagger } = \int d^{2}{\vec{x}} \, \psi ^H_{mn}({\vec{x}}) \, {\hat{a}}^{\dagger } ({\vec{x}}) \end{aligned}$$

are the creation operators of the eigenmodes of the interaction Hamiltonian, which are in the ${\text{HG}}_{mn}$ modes $\psi ^H_{mn}({\vec{x}})$. The operators satisfy the bosonic commutation relation $[{\hat{A}}^{H}_{mn},\left( {\hat{A}}^{H}_{kl}\right) ^{\dagger }] = \delta _{mn,kl}$, constructing an orthonormal HG mode basis. Note that the Hamiltonian is a direct sum of $\left( {\hat{A}}^{H}_{mn}\right) ^{\dagger }\left( {\hat{A}}^{H}_{mn}\right) ^{\dagger } + {\hat{A}}^{H}_{mn} {\hat{A}}^{H}_{mn}$ from different ${\hbox{HG}}_{{mn}}$ modes, which means that it generates squeezed light in each of the multiple spatial modes. The eigenvalues $\mu ^{m+n}$ are associated with the relative squeezing levels in different modes, which will be discussed in the following section.

### Self-imaging OPO

Let us construct a cavity that is compatible with the spatially multimode Hamiltonian in Eq. (7). While a typical cavity resonates on a single ${\hbox{HG}}_{{00}}$ mode^13^, for our purpose, we require a degenerate cavity resonant on all ${\hbox{HG}}_{{mn}}$ modes. The cavity degeneracy is determined by the Gouy phase shift ($\theta _{G}$) accumulated by the ${\hbox{HG}}_{{00}}$ mode along one cavity round-trip^31,32^. A confocal cavity has $\theta _{G}=\pi \, {\mathrm{mod}}(2\pi )$, which is resonant only on half of ${\hbox{HG}}_{{mn}}$ modes having either an even or an odd number of $m+n$^18^. On the other hand, a self-imaging cavity exhibits $\theta _{G}=0 \, {\mathrm{mod}}(2\pi )$: it is a fully resonant cavity for all ${\hbox{HG}}_{{mn}}$ modes^21,22^.

**Figure 1:**
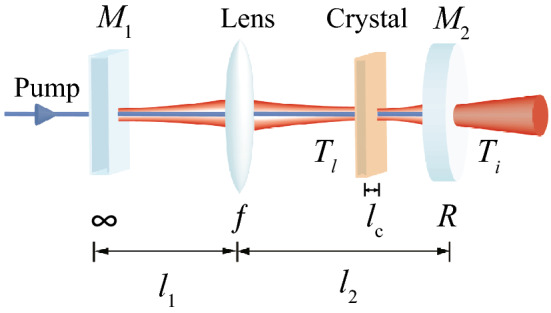
Self-imaging OPO for generating multimode squeezed light. $M_{1}$ is a perfect dichroic mirror transmitting the pump and reflecting the squeezed light, and $M_{2}$ is a partially reflective mirror having a transmittance of $T_{i}$ for the squeezed light. $T_{l}$ is the transmittance associated with the intracavity loss. The distances between the mirrors and the lens are $l_{1}$ and $l_{2}$, as represented in Eq. (9), where detunings $\Delta l_{1}$ and $\Delta l_{2}$ are required for stable operation of the OPO^22^.

Figure 1 describes a self-imaging OPO, which consists of a plane mirror $M_{1}$, a lens of focal length *f*, a nonlinear crystal of length $l_{c}$, and a curved mirror $M_{2}$ of a radius of curvature *R*. The lengths $l_{1}$ and $l_{2}$ can be expressed as

9 $$\begin{aligned} l_{1}&= f+f^{2}/R+\Delta l_{1} \nonumber \\ l_{2}&= f+R+\Delta l_{2}. \end{aligned}$$

When $\Delta l_{1} = \Delta l_{2} = 0$, the OPO becomes fully degenerate for all ${\hbox{HG}}_{{mn}}$ modes. This ideal condition, however, leads to cavity instability^22^, and thus, small detunings ($\Delta l_{1}, \Delta l_{2}$) are required for stable operation. The Gouy phase shift with such a detuning is

10 $$\begin{aligned} \theta _{G} = \cos ^{-1} \left( 1 + 2 {\Delta l_{2} \over R} \left( {\Delta l_{1} \over R} + {\Delta l_{2} \over R} - {\Delta l_{1} \over R} {\Delta l_{2} \over R} \right) \right) , \end{aligned}$$

where we have assumed $f=R$. As a result, when the cavity is locked for ${\hbox{HG}}_{{00}}$ mode, a high-order ${\hbox{HG}}_{{mn}}$ mode attains a phase shift of $(m+n)\theta _{G}$^31^. The cavity has HG eigenmodes

11 $$\begin{aligned} \psi _{mn}(x,y) = {\exp \left( -{x^{2}+y^{2} \over w_{c}^{2}}\right) H_m \left( {\sqrt{2}x \over w_{c}}\right) H_n\left( {\sqrt{2}y \over w_{c}}\right) \over w_{c} \sqrt{2^{m+n-1}\pi m! n!}} \end{aligned}$$

with the waist size of $w_{c} = \sqrt{R\lambda _0/2\pi }$ ($\lambda _0$: the free space wavelength) for small detunings $\Delta l_{1}, \Delta l_{2} \ll R$, and the associated creation operators are

12 $$\begin{aligned} {\hat{A}}_{mn}^\dagger = \int d^{2}{\vec{x}} \, \psi _{mn}({\vec{x}}) \, {\hat{a}}^{\dagger } ({\vec{x}}). \end{aligned}$$

To match the cavity modes with the eigenmodes of the Hamiltonian in Eq. (4), we position the crystal at the cavity waist and set $R=2\pi w_{H}^{2} / \lambda _0$, while a more general case of mismatch between the modes will be discussed in “Effect of mode mismatch inside the OPO” section. In this configuration, we obtain a decoupled quantum-Langevin-equation for each ${\hat{A}}_{mn}$^33^,

13 $$\begin{aligned} {d \over dt}{\hat{A}}_{mn}(t) = {1 \over i\hbar }[{\hat{A}}_{mn}(t),{\hat{H}}] -\left( \gamma _{i}+\gamma _{l}-i\Delta _{mn}\right) {\hat{A}}_{mn}(t) +\sqrt{2\gamma _{i}}{\hat{A}}^i_{mn}(t) + \sqrt{2{\gamma }_{l}} {\hat{A}}_{mn}^l (t), \end{aligned}$$

where ${\hat{A}}_{mn}^i$ and ${\hat{A}}_{mn}^l$ are the annihilation operators of the input and the intra-cavity loss modes, respectively, and the corresponding decay rates are given by $\gamma _{i}=T_{i}/2\tau$ and $\gamma _{l} = T_{l}/2\tau$ ($T_{i}$ and $T_{l}$ are shown in Fig. 1, $\tau$: the round trip time). As we consider a cavity locked for the ${\hbox{HG}}_{{00}}$ mode, the cavity detuning frequency of ${\hbox{HG}}_{{mn}}$ mode, $\Delta _{mn}$, is given by $(m+n)\theta _{G}/\tau$. We have used the approximation $T_{i}, T_{l}, \theta _{G} \ll 1$. Using Eq. (7),

14 $$\begin{aligned} {1 \over i\hbar }[{\hat{A}}_{mn}(t),{\hat{H}}] = -g \mu ^{m+n} {\hat{A}}_{mn}^{\dagger }(t), \end{aligned}$$

and then, the Fourier transform of Eq. (13) becomes

15 $$\begin{aligned} (\gamma _{i}+\gamma _{l}-i\omega -i\Delta _{mn}) {\hat{A}}_{mn}(\omega ) + g \mu ^{m+n} {\hat{A}}^{\dagger }_{mn}(-\omega ) = \sqrt{2\gamma _{i}}{\hat{A}}^{i}_{mn}(\omega ) +\sqrt{2\gamma _{l}}{\hat{A}}^l_{mn}(\omega ) \end{aligned}$$

at frequency $$\omega$$.

To investigate quantum correlations of the generated light at sidebands frequency $\omega$, we employ a vector of quadrature operators in ${\hbox{HG}}_{{mn}}$ modes,

16 $$\begin{aligned} {\hat{\mathbf{Q}}}(\omega ) = [{\hat{X}}_{00}(\omega ),{\hat{X}}_{01}(\omega ),\ldots ,{\hat{P}}_{00}(\omega ),{\hat{P}}_{01}(\omega ),\ldots ]^T, \end{aligned}$$

where ${\hat{X}}_{mn}(\omega ) = {\hat{A}}_{mn}(\omega )+{\hat{A}}_{mn}^{\dagger }(-\omega )$ and ${\hat{P}}_{mn}(\omega ) = ({\hat{A}}_{mn}(\omega )-{\hat{A}}_{mn}^{\dagger }(-\omega ))/i$. Express Eq. (15) with this vector operator as

17 $$\begin{aligned} {\mathbf{M}}(\omega ){\hat{\mathbf{Q}}}(\omega ) = \sqrt{2\gamma _{i}}{\hat{\mathbf{Q}}}^{i}(\omega ) +\sqrt{2\gamma _{l}} {\hat{\mathbf{Q}}}^{l}(\omega ) \end{aligned}$$

with

18 $$\begin{aligned} {\mathbf{M}}(\omega ) = \left[ \begin{matrix} (\gamma _{i}+\gamma _{l} -i\omega ){\mathbf{I}} + {\mathbf{G}} &{} \quad {\mathbf{D}} \\ -{\mathbf{D}} &{}\quad (\gamma _{i}+\gamma _{l} -i\omega ){\mathbf{I}} - {\mathbf{G}}\\ \end{matrix} \right] , \end{aligned}$$

where ${\mathbf{I}}_{kl,mn}=\delta _{kl,mn}, {\mathbf{G}}_{kl,mn} = g\mu ^{k+l}\,\delta _{kl,mn}, {\mathbf{D}}_{kl,mn} = \Delta _{kl}\,\delta _{kl,mn}$. Using the input–output relation at the coupler ${\hat{\mathbf{Q}}}^i+{\hat{\mathbf{Q}}}^o = \sqrt{2\gamma _{i}}{\hat{\mathbf{Q}}}$ (${\hat{\mathbf{Q}}}^i$ and ${\hat{\mathbf{Q}}}^o$ are the quadrature vectors of input and output modes), Eq. (17) becomes

19 $$\begin{aligned} {\hat{\mathbf{Q}}}^o(\omega )=(2\gamma _{i}{\mathbf{M}}^{-1}(\omega )-{\mathbf{I}}){\hat{\mathbf{Q}}}^i(\omega )+2\sqrt{\gamma _{i} \gamma _{l}}{\mathbf{M}}^{-1}(\omega ){\hat{\mathbf{Q}}}^l(\omega ). \end{aligned}$$

As the input is the vacuum state, we obtain the covariance matrix generated from the OPO as follows

20 $$\begin{aligned} {\mathbf{V}}(\omega ) = 4\gamma _{i} \gamma _{l} {\mathbf{M}}^{-1}(\omega )({\mathbf{M}}^{-1}(-\omega ))^T + (2\gamma _{i}{\mathbf{M}}^{-1}(\omega )-{\mathbf{I}})(2\gamma _{i}{\mathbf{M}}^{-1}(-\omega )-{\mathbf{I}})^T. \end{aligned}$$

Note that the covariance matrix ${\mathbf{V}}(\omega )$ does not exhibit any coupling between different ${\hbox{HG}}_{{mn}}$ modes. We can therefore characterize it by considering each ${\hbox{HG}}_{{mn}}$ mode individually. The quantum state in each ${\hbox{HG}}_{{mn}}$ mode turns out to be a squeezed vacuum aligned with rotated quadratures ${\hat{X}}_{mn}^{(\Theta )}(\omega ), {\hat{P}}_{mn}^{(\Theta )}(\omega )$:

21 $$\begin{aligned} {\hat{X}}_{mn}^{(\Theta )}(\omega )&={\hat{X}}_{mn}(\omega ) \cos \Theta + {\hat{P}}_{mn}(\omega ) \sin \Theta \nonumber \\ {\hat{P}}_{mn}^{(\Theta )}(\omega )&= - {\hat{X}}_{mn}(\omega ) \sin \Theta + {\hat{P}}_{mn}(\omega ) \cos \Theta \nonumber \\ \langle \Delta ^{2} {\hat{X}}_{mn}^{(\Theta )}(\omega )\rangle&= 1 - \eta { {4 \left| {{\tilde{g}}_{mn}}\right| } \over { 2 \left| {{\tilde{g}}_{mn}}\right| + \sqrt{({\tilde{\omega }}^{2}-{\tilde{\Delta }}_{mn}^{2}+{\tilde{g}}_{mn}^{2}+1)^{2} +4 {\tilde{\Delta }}_{mn}^{2}}}} \nonumber \\ \langle \Delta ^{2} {\hat{P}}_{mn}^{(\Theta )}(\omega )\rangle&= 1 - \eta { {4 \left| {{\tilde{g}}_{mn}}\right| } \over { 2 \left| {{\tilde{g}}_{mn}}\right| - \sqrt{({\tilde{\omega }}^{2}-{\tilde{\Delta }}_{mn}^{2}+{\tilde{g}}_{mn}^{2}+1)^{2} +4 {\tilde{\Delta }}_{mn}^{2}}}} \nonumber \\ \langle \{ \Delta {\hat{X}}_{mn}^{(\Theta )}(\omega ), \Delta {\hat{P}}_{mn}^{(\Theta )}(\omega ) \} \rangle&= 0 \nonumber \\ \tan \Theta&= {2 {\tilde{\Delta }}_{mn} \over ({\tilde{\omega }}^{2}-{\tilde{\Delta }}_{mn}^{2}+ {\tilde{g}}_{mn}^{2} +1) +{\text{sgn}}[{\tilde{g}}_{mn}] \sqrt{({\tilde{\omega }}^{2}-{\tilde{\Delta }}_{mn}^{2}+ {\tilde{g}}_{mn}^{2} +1)^{2} + 4 {\tilde{\Delta }}_{mn}^{2}}}, \end{aligned}$$

where all the parameters are real numbers, and

22 $$\begin{aligned} \eta&= \frac{\gamma _{i}}{\gamma _{i}+\gamma _{l}} = \frac{T_{i}}{T_{i}+T_{l}} \nonumber \\ {\tilde{\omega }}&= \frac{\omega }{\gamma _{i}+\gamma _{l}} \nonumber \\ {\tilde{g}}_{mn}&= \frac{g}{\gamma _{i}+\gamma _{l}} \mu ^{m+n} \nonumber \\ {\tilde{\Delta }}_{mn}&= \frac{\Delta _{mn}}{\gamma _{i}+\gamma _{l}} = \frac{2\theta _{G}}{T_{i}+T_{l}} (m+n). \end{aligned}$$

Note that $\langle \Delta ^{2} {\hat{X}}_{mn}^{(\Theta )}(\omega )\rangle \le 1$ and $\langle \Delta ^{2} {\hat{P}}_{mn}^{(\Theta )}(\omega )\rangle \ge 1$, and as ${\tilde{\Delta }}_{mn} \rightarrow 0$, the angle $\Theta$ is 0 if $0 \le {\tilde{g}}_{mn}$ or $\pi /2$ if ${\tilde{g}}_{mn} < 0$. Without loss of generality, we will focus on the case of $0 \le {\tilde{g}}_{mn}$ by setting $0 \le g$ and $0 \le \mu < 1$ (equivalently, $0 < \xi \le 1$). One can further note that the intracavity loss $T_{l}$ makes reduction on the cavity escape efficiency $\eta$.

**Figure 2:**
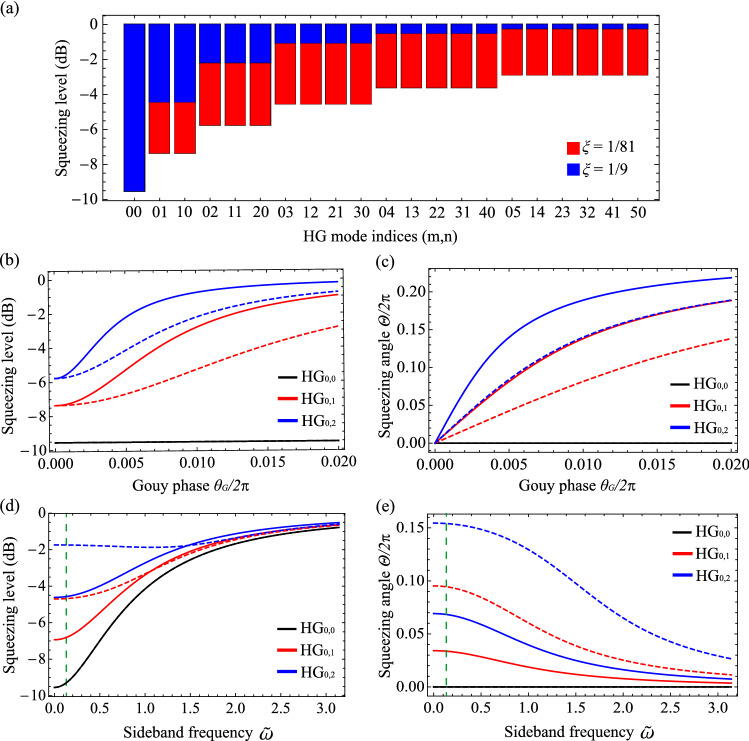
(**a**) Squeezing levels of multimode squeezed light for different focusing parameters of $\xi = 1/9$ and $\xi = 1/81$, when $T_{i} = 0.1, T_{l} = 0, {\tilde{\omega }} = 0, {\tilde{g}}_{00}=1/2$, and $\theta _{G} = 0$. In both cases, the squeezing levels in ${\hbox{HG}}_{{00}}$ are 9.5 dB. Effect of the Gouy phase shift $\theta _{G}$ on the squeezing levels in (**b**) and the squeezing angles in (**c**) for different ${\hbox{HG}}_{{mn}}$ modes and the transmittance $T_{i}$ of $M_{2}$. The solid lines are for $T_{i} = 0.1$, and the dashed lines are for $T_{i} = 0.2$. We use $\xi = 1/81$, $T_{l}=0$, ${\tilde{\omega }} =0$, and ${\tilde{g}}_{00} = 1/2$. Dependence of the squeezing levels in (**d**) and the squeezing angles in (**e**) on the sidebands frequency ${\tilde{\omega }}$. The solid and dashed lines are for $\theta _{G}/2\pi = 0.002$ and $\theta _{G}/2\pi = 0.006$, respectively. We use $\xi = 1/81$, $T_{i} = 0.1$, $T_{l}=0$, and ${\tilde{g}}_{00} = 1/2$. For the later analyses in Figs. (5, 6, 8), sideband frequency of ${\tilde{\omega }} = \pi /25$ will be used, which is indicated as the vertical dashed lines.

The generated multimode light from the OPO is therefore a collection of individual squeezed vacua in multiple ${\hbox{HG}}_{{mn}}$ modes, whose modal structure and quantum correlations are described by Eqs. (5) and (21), respectively. In more detail, the spectrum of quantum correlations is determined by the focusing parameter $\xi$, modifying ${\tilde{g}}_{mn}$ in Eq. (22), and the waist size is by $\xi$ and the pump waist $w_{p}$, as discussed in Eq. (5). For $\xi = 1$, making $\mu = 0$, the generated light is a single-mode squeezed vacuum: ${\hbox{HG}}_{{00}}$ contains a squeezed vacuum ($\langle \Delta ^{2} {\hat{X}}_{00}^{(\Theta )}(\omega )\rangle < 1$), but all high-order ${\hbox{HG}}_{{mn}}$ modes ($m,n\ne 0$) are vacuum states ($\langle \Delta ^{2} {\hat{X}}_{mn}^{(\Theta )}(\omega )\rangle = 1$). On the other hand, as $\xi$ becomes smaller than one, $\mu$ becomes positive, and high-order modes also exhibit squeezing ($\langle \Delta ^{2} {\hat{X}}_{mn}^{(\Theta )}(\omega )\rangle < 1$). Figure 2a compares the squeezing levels $\langle \Delta ^{2} {\hat{X}}_{mn}^{(\Theta )}(\omega )\rangle$ for different values of $\xi$. The smaller $\xi$ exhibits higher squeezing levels than the larger one for all high orders of ${\hbox{HG}}_{{mn}}$, and the associated Schmidt numbers calculated from Eq. (6) are 8.3 ($\xi =1/9$) and 20.7 ($\xi =1/81$). The angles $\Theta$ for ${\hat{X}}_{mn}^{(\Theta )}(\omega )$ quadratures are all zero as expected.

Figure 2b,c show the effects of the Gouy phase shift $\theta _{G}$ on the generated light. The Gouy phase creates the detuning ${\tilde{\Delta }}_{mn}$, which affects both the squeezing level and the squeezing angle. For small detunings, the squeezing level and the squeezing angle remain similar to the ideal self-imaging case, but as $\theta _{G}$ increases, the squeezing level $\langle \Delta ^{2} {\hat{X}}_{mn}^{(\Theta )}(\omega )\rangle$ gradually degrades to zero, and the squeezing angle $\Theta$ increases to $\pi /2$. Such effects are stronger for a smaller transmittance $T_{i}$ and a higher ${\hbox{HG}}_{{mn}}$ mode, as expected from Eq. (22). In addition, the squeezing level and the squeezing angle depend on the sideband frequency ${\tilde{\omega }}$, as shown in Fig. 2d,e. As ${\hbox{HG}}_{{00}}$ mode exhibits ${\tilde{\Delta }}_{mn}=0$, it behaves as a common OPO, where the squeezing level decreases while the squeezing angle remains constant as ${\tilde{\omega }}$ increases. On the other hand, for higher modes where ${\tilde{\Delta }}_{mn} \ne 0$, both of the squeezing level and the squeezing angle depend on the sideband frequency ${\tilde{\omega }}$. The rotated squeezing angle due to non-zero ${\tilde{\Delta }}_{mn}$ returns to zero as ${\tilde{\omega }}$ increases. The squeezing level, in most cases, gradually decreases to zero by increasing ${\tilde{\omega }}$, but there is a special case showing a non-monotonic behavior (the blue dashed line for ${\hbox{HG}}_{{02}}$ in Fig. 2d), which is because a non-zero value of ${\tilde{\omega }}$ makes the minimum value for $\langle \Delta ^{2} {\hat{X}}_{mn}^{(\Theta )}(\omega )\rangle$: such a case can take place at ${\tilde{\omega }} = \sqrt{{\tilde{\Delta }}_{mn}^{2} - {\tilde{g}}_{mn}^{2}-1}$ for ${\tilde{\Delta }}_{mn}^{2} > {\tilde{g}}_{mn}^{2}+1$, which can be derived from Eq. (21).

## Robustness on spatial mode mismatch

Spatial mode mismatch occurs when the mode of quantum light is different from a target mode, e.g. due to beam displacement, tilting, and beam size difference. Mode mismatch is especially detrimental for couplings with single-mode elements and processes, e.g., optical cavities, optical fibers, frequency conversion, and homodyne detection. In this section, we will show that the multimode squeezed light from the self-imaging OPO is robust on various types of spatial mode mismatch. When deriving the result, we will consider mode mismatch only in the *x*-direction, but the same result can be equally obtained for the *y*-direction because of the symmetry of the multimode squeezed light described in Eqs. (4,5,21).

### Mode-mismatch model

**Figure 3:**
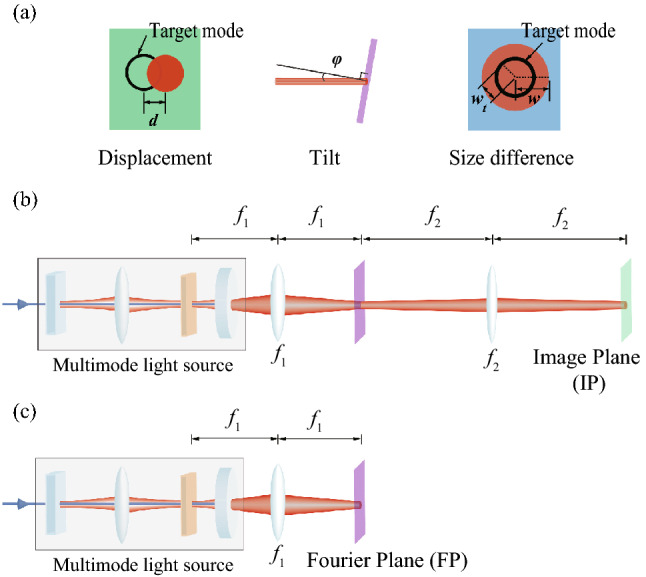
(**a**) Types of spatial mode mismatch. The displacement and the tilt are drawn in the *x*-direction for clarity, but they can be generalized to arbitrary directions. (**b**) 4f system for transformation into the image plane (IP). Two lenses of focal lengths $f_{1}$ and $f_{2}$ are used. (**c**) Transformation into the Fourier plane (FP) by employing a single lens (focal length: $f_{1}$).

To model the spatial mode mismatch, instead of fixing a target mode and varying the modes of quantum light, we will use an equivalent way for the simplicity of mathematical description: *we fix the quantum light but make deviations on the target mode.* We consider a target mode of ${\hbox{HG}}_{{00}}$ with the waist size of $w_{t}$

23 $$\begin{aligned} \phi _{00} (x,y) = \sqrt{2 \over \pi }{1 \over w_{t}} \exp {\left( -{x^{2} +y^{2} \over w_{t}^{2}}\right) }, \end{aligned}$$

and its deviations due to mode mismatches [displacement (*d*), tilt ($\varphi$), and size difference (*w*)] are

24 $$\begin{aligned} \phi ^{\mathrm{disp}} (x,y;d)&= \sqrt{2 \over \pi }{1 \over w_{t}} \exp {\left( -{(x-d)^{2} +y^{2} \over w_{t}^{2}}\right) }, \nonumber \\ \phi ^{\mathrm{tilt}} (x,y;\varphi )&= \sqrt{2 \over \pi }{1 \over w_{t}} \exp \left( -{x^{2}+y^{2} \over w_{t}^{2}} + i {2\pi \over \lambda _0} x \sin \varphi \right) , \nonumber \\ \phi ^{\mathrm{size}} (x,y;w)&= \sqrt{2 \over \pi }{1 \over w} \exp {\left( -{x^{2} +y^{2} \over w^{2}}\right) }, \end{aligned}$$

respectively. Figure 3a describes the mode mismatches on a target plane. One can expand a mismatched mode $\phi ^{\mathrm{mis}}$ based on the HG modes $\phi _{mn}$ stemming from Eq. (23)

25 $$\begin{aligned} \phi ^{\mathrm{mis}} (x,y;p) = \sum _{mn} \beta _{mn}^{\mathrm{mis}}(p)\, \phi _{mn} (x,y), \end{aligned}$$

where ${\mathrm{mis}} \in \{ {\text{disp,tilt,size}} \}$ and a mode-mismatching parameter $p \in \{ d, \varphi , w \}$, and

26 $$\begin{aligned} \beta _{mn}^{\mathrm{disp}} (d)&= {\delta _{n,0} \over \sqrt{m!}} \left( {d \over w_{t}}\right) ^m \exp \left( -{d^{2} \over 2w_{t}^{2}}\right) , \nonumber \\ \beta _{mn}^{\mathrm{tilt}} (\varphi )&= i^{m+n} {\delta _{n,0} \over \sqrt{m!}} \left( {\pi w_{t} \sin \varphi \over \lambda _0}\right) ^m \exp \left( -{\pi ^{2} w_{t}^{2} \sin ^{2} \varphi \over 2 \lambda _0^{2}}\right) , \nonumber \\ \beta _{mn}^{\mathrm{size}} (w)&= {\left\{ \begin{array}{ll} 0 &{}\quad n { \text{ or } } m {\text{: } \text{ odd }} \\ {\sqrt{m!n!}\,\left( {1\over 2}\tanh (\ln {w \over w_{t}})\right) ^{m+n \over 2} \over {m \over 2}!{n \over 2}! \cosh ( \ln {w \over w_{t}})}&{}\quad n { \text{ and } } m {\text{: } \text{ even }} \end{array}\right. }, \end{aligned}$$

and

27 $$\begin{aligned} \phi _{mn} (x,y) ={ \exp \left( {-{{x^{2}+y^{2}} \over w_{t}^{2}} }\right) H_m\left( {\sqrt{2}x \over w_{t}}\right) H_n\left( {\sqrt{2}y \over w_{t}}\right) \over {w_{t} \sqrt{2^{m+n-1} \pi m!n!} } }. \end{aligned}$$

**Figure 4:**
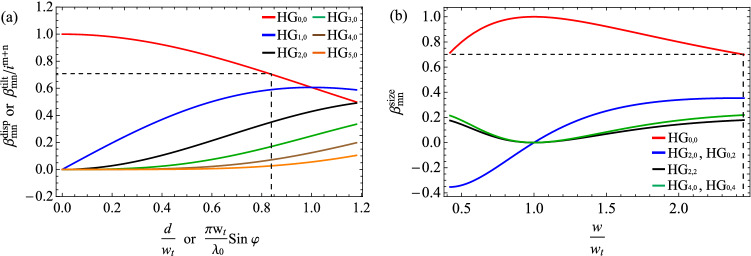
Coefficients arising from mode mismatch: displacement ($\beta _{mn}^{\mathrm{disp}}$) and tilt ($\beta _{mn}^{\mathrm{tilt}}$) in (**a**) and size difference ($\beta _{mn}^{\mathrm{size}}$) in (**b**). As mode mismatch increases, the contribution of ${\hbox{HG}}_{{00}}$ decreases gradually while high-order ${\hbox{HG}}_{{mn}}$ contribute more. Dashed lines represent reduction of contribution from ${\hbox{HG}}_{{00}}$ by 50 % (or $1/\sqrt{2}$ for the coefficient) due to mode mismatch, which takes place at $d/w_{t} = \pi w_{t} \sin \varphi /\lambda _0 = 0.83$ and $w/w_{t} = 2.45$.

Figure 4 shows the coefficients $\beta _{mn}^{\mathrm{disp}}$, $\beta _{mn}^{\mathrm{tilt}} (-i)^{m+n}$, and $\beta _{mn}^{\mathrm{size}}$, which are all real values. As $d, \varphi , {\text{ and }} w$ deviate from the ideal mode-matching condition more, ${\hbox{HG}}_{{00}}$ contributes less, which is replaced by the contributions from high-order ${\hbox{HG}}_{{mn}}$ modes.

By defining the creation operator $({\hat{B}}^{\mathrm{mis}})^\dagger$ for mode $\phi ^{\mathrm{mis}}$ and ${\hat{B}}^\dagger _{mn}$ for mode $\phi _{mn}$, Eq. (25) can be expressed as

28 $$\begin{aligned} ({\hat{B}}^{\mathrm{mis}})^\dagger = \sum _{mn} \beta _{mn}^{\mathrm{mis}}\, {\hat{B}}^\dagger _{mn}. \end{aligned}$$

The effect of mode mismatch can therefore be understood as contributions from high-order ${\hbox{HG}}_{{mn}}$ modes due to the emergence of non-zero coefficients $\beta _{mn}^{\mathrm{mis}}$. More specifically, a quadrature operator for the mismatched mode is written as

29 $$\begin{aligned} {\hat{B}}^{\mathrm{mis}} + \big ({\hat{B}}^{\mathrm{mis}}\big )^{\dagger } = \sum _{mn} {\text{Re}}\left[\beta _{mn}^{\mathrm{mis}}\right]\big ({\hat{B}}_{mn} + {\hat{B}}^{\dagger }_{mn}\big ) + {\text{Im}}\left[\beta _{mn}^{\mathrm{mis}}\right] \big ({\hat{B}}_{mn} - {\hat{B}}^{\dagger }_{mn}\big )/i. \end{aligned}$$

When the coefficients are real ($\beta _{mn} \in {\mathbb{R}}$) and no correlation exists between different ${\hbox{HG}}_{{mn}}$ and ${\hbox{HG}}_{{kl}}$, i.e., $\langle \Delta ({\hat{B}}_{mn} + {\hat{B}}^{\dagger }_{mn}) \Delta ({\hat{B}}_{kl} + {\hat{B}}^{\dagger }_{kl}) \rangle = 0$, the quadrature variance in the mismatched mode is

30 $$\begin{aligned} \left\langle \Delta ^{2} \left( {\hat{B}}^{\mathrm{mis}} + \big ({\hat{B}}^{\mathrm{mis}}\big )^{\dagger } \right) \right\rangle = \sum _{mn} \, \beta _{mn}^{2} \, \left\langle \Delta ^{2} \left( {\hat{B}}_{mn} + {\hat{B}}^{\dagger }_{mn} \right) \right\rangle , \end{aligned}$$

which is the weighted mean of the quadrature variances in the ${\hbox{HG}}_{{mn}}$ modes with the weighting factors of $\beta _{mn}^{2}$. As the mode mismatch increases, the weight for ${\hbox{HG}}_{{00}}$ decreases, and the noises from high-order ${\hbox{HG}}_{{mn}}$ come in. Since single-mode squeezed light exhibits a squeezed noise in ${\hbox{HG}}_{{00}}$ and the vacuum noise in ${\hbox{HG}}_{{mn}}$, the squeezing level quickly degrades to the vacuum noise due to mode mismatch. On the other hand, multimode squeezed light exhibits squeezed noises in high-order ${\hbox{HG}}_{{mn}}$ modes together. As a result, multimode light can show less degradation on the squeezing level, which, therefore, tolerates more mode mismatch than single-mode light does.

### Mode-mismatch tolerance of multimode squeezed light

We will use the multimode squeezed light in “Quantum properties of multimode squeezed light” section to investigate its robustness on mode mismatch. We first consider the ideal mode matching of the multimode light with a target mode and then, to account for mode mismatch, we will make deviations on the target mode, as discussed in “Mode-mismatch model” section. Figure 3b,c depicts linear optical elements through which the multimode light propagate from the OPO to a target plane. The optical elements transform the HG modes $\psi _{mn}$ in Eq. (11) into new modes ${\mathscr {I}} [\psi _{mn}]$, which can be obtained by Huygen-Fresnel’s integral ${\mathscr {I}}$ through the associated ABCD matrix^31^:

31 $$\begin{aligned} {\mathscr {I}} [\psi _{mn}] ={ 1 \over {w_{1} \sqrt{2^{m+n-1} \pi m!n!} } } \left( {w_{1} \over A w_{c}+2i B/k w_{c}}\right) ^{m+n+1} H_m \left( {\sqrt{2} x \over w_{1}}\right) H_n \left( {\sqrt{2}y \over w_{1}}\right) \exp \left( {i k{{x^{2}+y^{2}} \over 2 q} }\right) , \end{aligned}$$

where *A*, *B*, *C*,  and *D* are the matrix elements, and

$$\begin{aligned} q&= {-A (i k w_{c}^{2} /2) + B \over -C (i k w_{c}^{2} /2) + D}, \\ w_{1}^{2}&= A^{2} w_{c}^{2} + (2 B/ k w_{c})^{2}. \end{aligned}$$

First, let us consider the transformation into the image plane (IP) in Fig. 3b. By choosing focal lengths satisfying ${f_{2} / f_{1}} = {w_{t} / w_{c}}$, the new modes become

32 $$\begin{aligned} {\mathscr {I}}^{\mathrm{IP}} [\psi _{mn}] = (-1)^{m+n+1} \phi _{mn} (x,y), \end{aligned}$$

which coincides with $\phi _{mn}$ in Eq. (27) with the additional phase factor $(-1)^{m+n+1}$. Denoting the unitary operation for ${\mathscr {I}}^{\mathrm{IP}}$ by ${\hat{U}}_{\mathrm{IP}}$, the associated creation operators show a simple relation

33 $$\begin{aligned} {\hat{A}}^\dagger _{mn} = (-1)^{m+n+1}\,{\hat{U}}_{\mathrm{IP}}^\dagger {\hat{B}}^\dagger _{mn} {\hat{U}}_{\mathrm{IP}}. \end{aligned}$$

Together with Eq. (28),

34 $$\begin{aligned} {\hat{U}}_{\mathrm{IP}}^\dagger ({\hat{B}}^{\mathrm{mis}})^\dagger {\hat{U}}_{\mathrm{IP}} = \sum _{mn} \beta _{mn}^{\mathrm{mis}}\,(-1)^{m+n+1}\, {\hat{A}}^\dagger _{mn}. \end{aligned}$$

We thus obtain the expression of a quadrature variance at the mismatched mode at sideband frequency $\omega$:

35 $$\begin{aligned}&\left\langle \Delta ^{2} \left( {\hat{U}}_{\mathrm{IP}}^\dagger {\hat{X}}^{\mathrm{mis}}(\omega ) {\hat{U}}_{\mathrm{IP}} \right) \right\rangle = {\mathbf{r}} ^ T {\mathbf{V}}(\omega ) {\mathbf{r}}, \end{aligned}$$

where the sideband quadrature operator ${\hat{X}}^{\mathrm{mis}}(\omega )$ is ${\hat{B}}^{\mathrm{mis}}(\omega ) + ({\hat{B}}^{\mathrm{mis}})^\dagger (-\omega )$, the covariance matrix ${\mathbf{V}}(\omega )$ is given in Eqs. (20,21), and

36 $$\begin{aligned} {\mathbf{r}}&= [\mathrm{Re}(\gamma _{00}),\ldots ,\mathrm{Im}(\gamma _{00}),\ldots ]^T \nonumber \\ \gamma _{mn}&=\beta _{mn}^{\mathrm{mis}}\,(-1)^{m+n+1}. \end{aligned}$$

As ${\mathbf{V}}(\omega )$ contains ${\hat{X}}$-quadrature squeezed vacua in ${\hbox{HG}}_{{mn}}$ modes when $\theta _{G} = 0$ and ${\tilde{g}}_{mn} > 0$, if $\gamma _{mn} \in {\mathbb{R}}$, only squeezed-quadrature noises are coupled into the mismatched mode, which makes the multimode squeezed light robust on mode mismatch. In the image plane, such a condition is satisfied for mode mismatches by displacement and beam-size difference,

37 $$\begin{aligned} \gamma _{mn}^{\mathrm{disp}}&= \beta _{mn}^{\mathrm{disp}} (-1)^{m+n+1} \in {\mathbb{R}} \nonumber \\ \gamma _{mn}^{\mathrm{size}}&= \beta _{mn}^{\mathrm{size}} (-1)^{m+n+1} \in {\mathbb{R}}. \end{aligned}$$

Second, we investigate the mode mismatch in the Fourier plane (FP), described in Fig. 3c. The focal length of the lens is chosen as $f_{1} = w_{t} w_{c} \pi / \lambda _0$. Denoting the unitary operation for transforming into the Fourier plane by ${\hat{U}}_{\mathrm{FP}}$, the associated creation operators are related as

38 $$\begin{aligned} {\hat{A}}^\dagger _{mn} = i^{m+n+1}\, {\hat{U}}_{\mathrm{FP}}^\dagger {\hat{B}}^\dagger _{mn} {\hat{U}}_{\mathrm{FP}}, \end{aligned}$$

and thus, the variance by the sideband operator ${\hat{X}}^{\mathrm{mis}}(\omega ) = {\hat{B}}^{\mathrm{mis}}(\omega ) + ({\hat{B}}^{\mathrm{mis}})^\dagger (-\omega )$ is

39 $$\begin{aligned}&\left\langle \Delta ^{2} \left( {\hat{U}}_{\mathrm{FP}}^\dagger {\hat{X}}^{\mathrm{mis}}(\omega ) {\hat{U}}_{\mathrm{FP}} \right) \right\rangle = {\mathbf{s}} ^ T {\mathbf{V}}(\omega ) {\mathbf{s}}, \end{aligned}$$

where

40 $$\begin{aligned} {\mathbf{s}}&= [\mathrm{Re}(\zeta _{00}),\ldots ,\mathrm{Im}(\zeta _{00}),\ldots ]^T \nonumber \\ \zeta _{mn}&=\beta _{mn}^{\mathrm{mis}}\,(-i)^{m+n+1}. \end{aligned}$$

Like the case of the image plane, the condition $\zeta _{mn} \in {\mathbb{R}}$ makes the multimode squeezed light robust on mode mismatch. In the Fourier plane, mismatches by tilt and beam-size difference with an additional $\pi /2$-phase shift satisfy the condition,

41 $$\begin{aligned} \zeta _{mn}^{\mathrm{tilt}}&= \beta _{mn}^{\mathrm{tilt}} (-i)^{m+n+1} e^{i \pi /2} \in {\mathbb{R}} \nonumber \\ \zeta _{mn}^{\mathrm{size}}&= \beta _{mn}^{\mathrm{size}} (-i)^{m+n+1} e^{i \pi /2} \in {\mathbb{R}}. \end{aligned}$$

**Figure 5:**
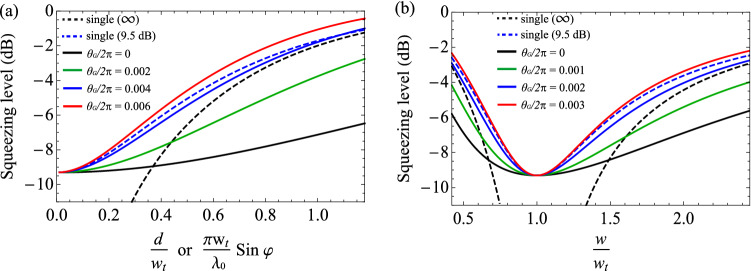
Robustness of multimode squeezed light on mode mismatch. The squeezing level coupled into a target mode is plotted by varying (**a**) displacement or tilt and (**b**) beam size. We use $T_{i} = 0.1$, $T_{l} = 0$, ${\tilde{\omega }} =\pi /25$, and ${\tilde{g}}_{00} = 1/2$. The black dashed line is for the single-mode infinitely squeezed light, and the blue dashed line is for single-mode 9.5-dB squeezed light, and the solid lines are for multimode squeezed light with $\xi = 1/81$ for different Gouy phase shifts of $\theta _{G}$.

Figure 5 shows the robustness of the multimode squeezed light ${\mathbf{V}}(\omega )$ on mode mismatch, compared with the result of a single-mode squeezed light in ${\hbox{HG}}_{{00}}$. We first consider the multimode light by the ideal self-imaging condition ($\theta _{G}/2\pi =0$, black solid line), and more general cases will be discussed later. As shown in Fig. 5a, when mode mismatch by displacement (in the image plane) or tilt (in the Fourier plane) occurs, the squeezing level by the single-mode light (original squeezing of 9.5 dB, blue dashed line) quickly degrades, e.g., less than 3 dB for $d/w_{t} > 1$ or $\pi w_{t} \sin \varphi / \lambda _0 > 1$. On the other hand, the multimode light with the same squeezing in ${\hbox{HG}}_{{00}}$ maintains the squeezing level very well by tolerating the mode mismatch, exhibiting more than 7 dB in the same condition. It is noteworthy that, at sufficiently large mode mismatch, the multimode light even outperforms single-mode light with infinite squeezing (black dashed line). Furthermore, the multimode squeezed light is robust on beam-size mismatch on both the image plane and the Fourier plane, as shown in Fig. 5b. Similar to the previous case, the multimode light maintains the squeezing level very well in the influence of mode mismatch, even outperforming the single-mode infinitely squeezed light.

### Effect of loss

**Figure 6:**
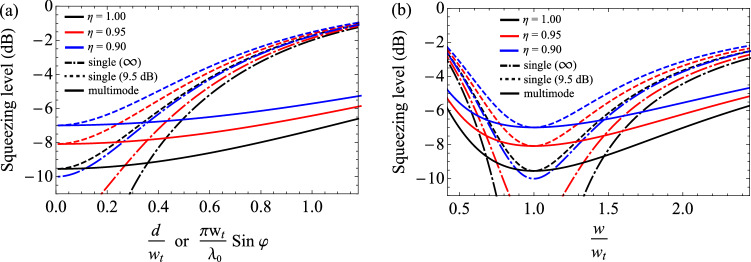
Effect of loss on the squeezing level with mode mismatch. $1-\eta$ corresponds to the total optical loss (e.g. by including the detection inefficiency). For different amounts of $\eta =1$ (black), $\eta =0.95$ (red), and $\eta =0.9$ (blue), the performances of three different squeezed lights are compared (dot dashed: single-mode infinitely squeezed light, dashed: single-mode 9.5-dB squeezed light, and solid: multimode squeezed light with $\xi = 1/81$). We use the following parameters for the plots: ${\tilde{\omega }} =\pi /25$, ${\tilde{g}}_{00} = 1/2$, ${\tilde{\Delta }}_{mn} = 0$.

Here we investigate the effect of loss on the multimode light in terms of the mode mismatch. In Eqs. (21,22), the escape efficiency $\eta$ accounts for the intracavity loss, but it can be generalized to incorporate the total loss in the system, $1-\eta$, e.g. propagation and detection losses. Figure 6 shows the squeezing level by mode mismatch for different amounts of losses. $\eta =1$ corresponds to no loss in the total system ($1-\eta =0$), which is identical with the black solid lines ($\theta _{G}/2\pi =0$) in Fig. 5a,b. As the loss increases by reducing $\eta$, the squeezing level decreases for all the three cases of infinitely squeezed single-mode light, single-mode squeezed light (9.5 dB), and the multimode light (9.5 dB in ${\hbox{HG}}_{{00}}$ mode). Although such losses exist, we still find that the multimode light is more robust on mode mismatch than the single-mode light (9.5 dB), and for a sufficiently large mismatch, it again outperforms the infinitely squeezed light.

### Effect of mode mismatch inside the OPO

In “Self-imaging OPO”, we assumed that the eigenmodes of the interaction Hamiltonian (4) perfectly match with the cavity modes (11), i.e., the same waist size, $w_{H} = w_{c}$. However, mode mismatch can take place inside the OPO due to waist size difference ($w_{H} \ne w_{c}$) or the Gaussian approximation (${\mathrm{sinc}}(x^2) \approx \exp (-\alpha x^{2})$) used for the Kernel. We investigate how the mode mismatch inside the OPO affects the robustness of multimode light on mode mismatch to a target mode.

**Figure 7:**
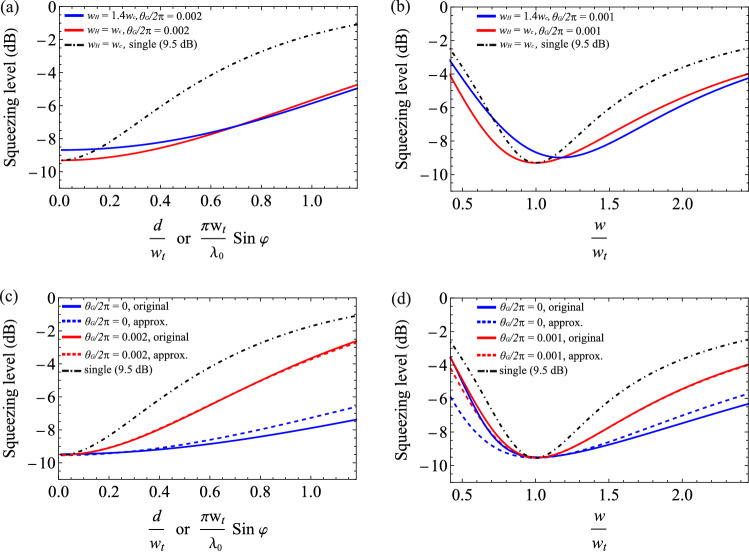
(**a**,**b**) Effect of mode mismatch due to difference in the cavity waist ($w_{c}$) and the interaction Hamiltonian waist ($w_{H}$). The squeezing level due to a large difference in waists ($w_{H} = 1.4 w_{c}$, solid blue) is compared with the case of no difference in waists ($w_{H} = w_{c}$, solid red) and with the single-mode 9.5-dB squeezed light (black dot dashed). (**c**,**d**) Effect of the Gaussian approximation (4) for the original interaction Hamiltonian (2,46). The solid lines are for the original Hamiltonian and the dashed lines are for the Gaussian approximation, which are plotted without a Gouy phase (blue) and with a Gouy phase (red). Black dot-dashed line is for the single-mode 9.5-dB squeezed light. We use the following parameters for the plots: $\xi = 1/81, T_{i} = 0.1$, $T_{l} = 0$, ${\tilde{\omega }} =\pi /25$, ${\tilde{g}}_{00} = 1/2$, ${\tilde{\Delta }}_{mn} = 0$.

At first, we consider the waist size difference ($w_{H} \ne w_{c}$) while keeping the Gaussian approximation. To deal with the size difference, we employ a change of basis from the eigenmodes of the interaction Hamiltonian to the cavity modes

42 $$\begin{aligned} \psi _{mn}(x,y) = \sum _{m',n'} {\mathbf{U}}_{mn,m'n'}(w_{c},w_{H}) \psi ^H_{m',n'}(x,y) \end{aligned}$$

where the basis change matrix ${\mathbf{U}}(w_{c},w_{H})$ is given as^34^

43 $$\begin{aligned} {\mathbf{U}}_{mn,m'n'}(w_{c},w_{H})&= {\left\{ \begin{array}{ll} {\sqrt{m! m'! n! n'!} \over \cosh ^{m+n+1} \left( \ln {w_{H} \over w_{c}}\right) } \left( {\tanh \left( \ln {w_{H} \over w_{c}}\right) \over 2}\right) ^{m'+n'-m-n\over 2} f\left( \ln {w_{H} \over w_{c}},m,m'\right) f\left( \ln {w_{H} \over w_{c}},n,n'\right) &{} \vert m-m'\vert \; {\text{and}} \; \vert n-n'\vert {\text{: even}} \\ 0 &{} {\text{else}} \end{array}\right. } \end{aligned}$$

44 $$\begin{aligned} f(r,m,m')&= \sum _{ {m-m'\over 2} \le k \le {m\over 2} } \, {(-1)^{k} \left( {\sinh r \over 2}\right) ^{2k} \over k!(m-2k)!\left( k+{m-m' \over 2}\right) }. \end{aligned}$$

By describing the interaction Hamiltonian in the cavity mode basis, one obtains a modified gain matrix $\mathbf{G'}$

45 $$\begin{aligned} \mathbf{G'} = {\mathbf{U}}(w_{c},w_{H})\,{\mathbf{G}}\,{\mathbf{U}}^{\dagger }(w_{c},w_{H}), \end{aligned}$$

where ${\mathbf{G}}$ is the original gain matrix in Eq. (18). Differently from ${\mathbf{G}}$, $\mathbf{G'}$ is a non-diagonal matrix in general. One can use $\mathbf{G'}$ instead of ${\mathbf{G}}$ for calculating the covariance matrix (20) and the squeezing levels in target modes (35,39).

Figure 7a,b shows that, even with a large difference in waist sizes ($w_{H} = 1.4 w_{c}$), the light from the self-imaging OPO still exhibits robustness on mode mismatch: displacement or tilt in Fig. 7a and beam size in Fig. 7b. This robustness is due to the multimode nature of the interaction Hamiltonian: although the waist size of the interaction Hamiltonian varies, the interaction Hamiltonian can still provide a multimode gain ($\mathbf{G'}$) in the multiple cavity modes, which in turn generates multimode squeezed light required for robustness on mode mismatch.

Second, we consider the interaction Hamiltonian without using the Gaussian approximation. Let us rewrite the associated kernel (2) using $w_{p}$, $\xi$, and $\alpha$:

46 $$\begin{aligned} {\tilde{K}}({\vec{q}}_{s},{\vec{q}}_{i}) = \exp \left( -{w_{p}^{2}\over 4} \vert {\vec{q}}_{s}+{\vec{q}}_{i}\vert ^{2}\right) {\mathrm{sinc}} \left( {w_{p}^{2} \over 4} {\xi \over \alpha } \vert {\vec{q}}_{s}-{\vec{q}}_{i}\vert ^{2}\right) . \end{aligned}$$

We decompose the kernel numerically since analytical expression is unknown due to the inclusion of the sinc function^28^. The Schmidt number solely depends on $\xi / \alpha$ because $w_{p}$ just acts as the scaling factors of ${\vec{q}}_{s}$ and ${\vec{q}}_{i}$. To compare the properties of the original Hamiltonian (2,46) and those by the approximated one (4), we find, for a given $\xi$, the coefficient $\alpha$ that gives the same Schmidt number as the Gaussian approximation (6); this way of choosing $\alpha$ is justified because the robustness on mode mismatch originates from the occupation of squeezed light in multiple modes, depending highly on the Schmidt number. For $\xi =1/81$, the corresponding $\alpha$ is 0.46. In addition, $w_{p}$ is determined by maximizing the overlap between the first eigenmode of Eq. (46) and the ${\hbox{HG}}_{{00}}$ cavity mode. A modified gain matrix $\mathbf{G'}$ is then obtained by

47 $$\begin{aligned} \mathbf{G'}_{mn,m'n'} = g\int d^{2}{\vec{x}}_{s} d^{2}{\vec{x}}_{i} \, K'({\vec{x}}_{s},{\vec{x}}_{i})\psi _{mn}({\vec{x}}_{s})\psi _{m'n'}({\vec{x}}_{i}), \end{aligned}$$

where $K'({\vec{x}}_{s},{\vec{x}}_{i})$ is the inverse Fourier transform of Eq. (46), and $\psi _{mn}(x,y)$ are the cavity modes defined in Eq. (11). $\mathbf{G'}$, being a non-diagonal matrix, is used instead of ${\mathbf{G}}$ to find the covariance matrix (20) and the squeezing levels in target modes (35,39).

In Fig. 7c,d, we compare the robustness of mode mismatch by the original Hamiltonian and by the approximated Hamiltonian. It is evident that both cases exhibit robustness on mode mismatch by outperforming the single-mode squeezed light. In Fig. 7c with $\theta _{G}/2\pi = 0$, one can find that the original Hamiltonian shows a slightly better performance than the approximated one. It is because, while the Schmidt numbers are the same, the eigenvalue spectrum of the original Hamiltonian is distributed more toward lower-order eigenmodes than that of the approximated Hamiltonian, which is advantageous for a small amount of mode mismatch. At $\theta _{G}/2\pi = 0.002$, their difference becomes negligible because of the squeezing angle rotation in high-order modes. In Fig. 7d with $\theta _{G}/2\pi = 0$, the original Hamiltonian shows better squeezing level for $w/w_{t} > 1$ but worse for $w/w_{t} < 1$ when compared with the approximated Hamiltonian: the asymmetry comes from the negative correlations between even-order ${\hbox{HG}}_{{mn}}$ modes for the original Hamiltonian (e.g., $\mathbf{G'}_{00,04}$, $\mathbf{G'}_{00,22} < 0$ in Eq. (47)). At $\theta _{G}/2\pi = 0.001$, the difference in the performances of the two Hamiltonians is negligible as in the case of Fig. 7c.

### Effect of Gouy phase shift

Until now, we have explored the robustness on mode mismatch in the ideal self-imaging condition. For stable operation of the OPO, however, a small detuning by the Gouy phase shift is necessary^22^. The detuning degrades the squeezing level and rotates the squeezing angle for high-order HG modes, as discussed in Fig. 2. Here we investigate whether the robustness on mode mismatch can still be sustained with detunings from the ideal condition.

Figure 5 compares the squeezing levels coupled in a target mode with non-zero Gouy phase shifts, $\theta _{G} / 2\pi = 0.002, 0.004$, and 0.006 for the displace/tilt mismatching and $\theta _{G} / 2\pi = 0.001, 0.002$, and 0.003 for the size mismatching. As expected, the squeezing level becomes degraded as more Gouy phase shift is introduced. For $\theta _{G} / 2\pi = 0.002$ in (a) and $\theta _{G} / 2\pi = 0.001$ in (b), the generated multimode light can still beat the performance of the infinitely squeezed single-mode light for a sufficiently large mismatch. The cases of $\theta _{G} / 2\pi = 0.004$ in (a) and $\theta _{G} / 2\pi = 0.002$ in (b) exhibit a squeezing level worse than the infinitely squeezed light but better than 9.5 dB squeezed single-mode light. However, we find no advantage in using the multimode light with $\theta _{G} / 2\pi = 0.006$ in (a) and $\theta _{G} / 2\pi = 0.003$ in (b), being worse than the 9.5 dB single-mode light; in this regime, due to the rotations of the squeezing angles of high-order HG modes in Fig. 2c, ${\hat{X}}_{mn}(\omega )$ quadratures exhibit larger noises than the vacuum noise. To take advantage of using multimode light, keeping a small-enough Gouy phase shift is required.

**Figure 8:**
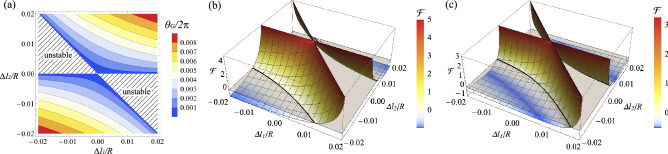
(**a**) Gouy phase shift ($\theta _{G}$) of the OPO with detunings $\Delta l_{1}$ and $\Delta l_{2}$. Hatched areas show the unstable regions. (**b**,**c**) Enhancement factor ${\mathscr {F}}$ of using multimode squeezed light, defined in Eq. (48). When mode mismatch is 50% by (**b**) displacement or tilt and by (**c**) size difference, we compare the squeezing levels in a target mode by single-mode squeezed light (9.5 dB) and multimode squeezed light (9.5 dB at ${\hbox{HG}}_{{00}}$ and $\xi = 1/81$). $\Delta l_{1}$ and $\Delta l_{2}$ are the detunings applied for cavity stability. ${\mathscr {F}}>0$ indicates that the multimode squeezed light is better than the single-mode light. The area where the graph is not drawn corresponds to an unstable region of the OPO. For the plot, we use parameters of $T_{i} = 0.1$, $T_{l} = 0$, ${\tilde{\omega }} =\pi /25$, and ${\tilde{g}}_{00} = 1/2$.

We, therefore, investigate how small Gouy phase shift is required to exhibit advantages of using multimode squeezed light. For the quantification, we define an enhancement factor ${\mathscr {F}}$ in decibels,

48 $$\begin{aligned} {\mathscr {F}} = -10 \log _{10} \left[ { \Delta ^{2} {\hat{X}}^{({\mathrm{mul}})} \over \Delta ^{2} {\hat{X}}^{({\mathrm{sin}})} } \right] , \end{aligned}$$

where $\Delta ^{2} {\hat{X}}^{({\mathrm{sin}})}$ is the squeezing level by single-mode squeezed light, and $\Delta ^{2} {\hat{X}}^{({\mathrm{mul}})}$ is the squeezing level by multimode squeezed light (having the same initial squeezing level in ${\hbox{HG}}_{{00}}$ as the single-mode light). A positive value of ${\mathscr {F}}$ indicates that the multimode squeezed light is more robust to mode mismatch than the single-mode squeezed light. As the Gouy phase shift is determined by the detuning ratios $\Delta l_{1} /R$ and $\Delta l_{2} /R$, as given in Eq. (10), we calculate the enhancement factor as varying the detunings. Figure 8 shows the enhancement factor of using the multimode squeezed light when the mode overlap by mismatch is 50% (corresponding to the dashed lines at $d/w_{t} = \pi w_{t} \sin \varphi /\lambda _0 =0.83$ and $w/w_{t}=2.45$ in Fig. 4). A broad range of $\Delta l_{1} /R$ and $\Delta l_{2} /R$ exhibits enhancements compared with the single-mode case. Comparing (a) and (b) in Fig. 8, mode mismatch by size difference requires more stringent conditions for the enhancement; this is because size difference involves higher-orders of HG modes than those by displacement and tilt, as shown in Fig. 4, and the squeezed lights in higher-order modes are more susceptible to the Gouy phase shift as shown in Fig. 2b,c. However, these stringent conditions are still achievable using only off-the-shelf positioning devices: a typical OPO employs a curved mirror with the radius of the curvature in the order of $R=100$ mm, and position controllability in the order of 100 $\upmu$m (e.g. by using a linear stage) can readily achieve very small values of $\Delta l_{1} /R, \Delta l_{2} /R = 0.001$.

## Conclusion

In this paper, we have shown that multimode squeezed light generated from a self-imaging OPO is robust on spatial mode mismatch. First, we found an analytic form of the quantum properties of the multimode light at sidebands frequency by taking into account the Gouy phase shift (required for OPO stability) and the intracavity loss. By decomposing mode mismatches of displacement, tilt, and size difference into a ${\hbox{HG}}_{{mn}}$ mode basis, we found that the mode mismatches induce contributions from high-order ${\hbox{HG}}_{{mn}}$ modes, which makes the multimode squeezed light robust on mode mismatch. We showed that the multimode light from the self-imaging OPO with a small Gouy phase shift can even outperform the single-mode infinitely squeezed light, in terms of displacement and size difference in the image plane and of tilt and size difference in the Fourier plane. Such robustness on the multiple cases of mode mismatch is made possible because of the fully degenerate nature of the self-imaging OPO, which cannot be accomplished by using a confocal OPO^18^ or two single-mode OPOs^16^. Our work of mitigating the mode mismatching loss will have broad applications to quantum technologies based on squeezed light, e.g., quantum-enhanced gravitational-wave detection^11,12^, deterministic quantum teleportation^7^, measurement-based quantum computing^1–4^, and Gaussian boson sampling^5,6^.

## References

[1] Asavanant W (2019). Generation of time-domain-multiplexed two-dimensional cluster state. Science (New York, NY).

[2] Larsen MV, Guo X, Breum CR, Neergaard-Nielsen JS, Andersen UL (2019). Deterministic generation of a two-dimensional cluster state. Science (New York, NY).

[3] Ra Y-S (2020). Non-Gaussian quantum states of a multimode light field. Nat. Phys..

[4] Pfister O (2020). Continuous-variable quantum computing in the quantum optical frequency comb. J. Phys. B At. Mol. Opt. Phys..

[5] Zhong H-S (2020). Quantum computational advantage using photons. Science (New York, NY).

[6] Arrazola JM (2021). Quantum circuits with many photons on a programmable nanophotonic chip. Nature.

[7] Liu S, Lou Y, Jing J (2020). Orbital angular momentum multiplexed deterministic all-optical quantum teleportation. Nat. Commun..

[8] Giovannetti V, Lloyd S, Maccone L (2004). Quantum-enhanced measurements: Beating the standard quantum limit. Science.

[9] Taylor MA (2013). Biological measurement beyond the quantum limit. Nat. Photonics.

[10] Guo X (2020). Distributed quantum sensing in a continuous-variable entangled network. Nat. Phys..

[11] Tse M (2019). Quantum-enhanced advanced LIGO detectors in the era of gravitational-wave astronomy. Phys. Rev. Lett..

[12] Acernese F (2019). Increasing the astrophysical reach of the advanced virgo detector via the application of squeezed vacuum states of light. Phys. Rev. Lett..

[13] Vahlbruch H, Mehmet M, Danzmann K, Schnabel R (2016). Detection of 15 dB squeezed states of light and their application for the absolute calibration of photoelectric quantum efficiency. Phys. Rev. Lett..

[14] Oelker E, Barsotti L, Dwyer S, Sigg D, Mavalvala N (2014). Squeezed light for advanced gravitational wave detectors and beyond. Opt. Express.

[15] Töyrä D (2017). Multi-spatial-mode effects in squeezed-light-enhanced interferometric gravitational wave detectors. Phys. Rev. D.

[16] Steinlechner S (2018). Mitigating mode-matching loss in nonclassical laser interferometry. Phys. Rev. Lett..

[17] Wittel, H. Active and passive reduction of high order modes in the gravitational wave detector GEO 600. PhD Thesis (2015).

[18] Lugiato LA, Grangier P (1997). Improving quantum-noise reduction with spatially multimode squeezed light. J. Opt. Soc. Am. B.

[19] Embrey CS, Turnbull MT, Petrov PG, Boyer V (2015). Observation of localized multi-spatial-mode quadrature squeezing. Phys. Rev. X.

[20] Hsu MTL, Delaubert V, Lam PK, Bowen WP (2004). Optimal optical measurement of small displacements. J. Opt. B Quantum Semiclass. Opt..

[21] Arnaud JA (1969). Degenerate optical cavities. Appl. Opt..

[22] Chalopin B, Chiummo A, Fabre C, Maître A, Treps N (2010). Frequency doubling of low power images using a self-imaging cavity. Opt. Express.

[23] Lopez L (2009). Multimode quantum properties of a self-imaging optical parametric oscillator: Squeezed vacuum and Einstein–Podolsky–Rosen-beams generation. Phys. Rev. A.

[24] Chalopin B, Scazza F, Fabre C, Treps N (2010). Multimode nonclassical light generation through the optical-parametric-oscillator threshold. Phys. Rev. A.

[25] Chalopin B, Scazza F, Fabre C, Treps N (2011). Direct generation of a multi-transverse mode non-classical state of light. Opt. Express.

[26] Caspani L, Brambilla E, Gatti A (2010). Tailoring the spatiotemporal structure of biphoton entanglement in type-I parametric down-conversion. Phys. Rev. A.

[27] Monken CH, Ribeiro PHS, Pádua S (1998). Transfer of angular spectrum and image formation in spontaneous parametric down-conversion. Phys. Rev. A.

[28] Straupe SS, Ivanov DP, Kalinkin AA, Bobrov IB, Kulik SP (2011). Angular Schmidt modes in spontaneous parametric down-conversion. Phys. Rev. A.

[29] Miatto FM, Brougham T, Yao AM (2012). Spatial Schmidt modes generated in parametric down-conversion. Eur. Phys. J. D.

[30] Law CK, Eberly JH (2004). Analysis and interpretation of high transverse entanglement in optical parametric down conversion. Phys. Rev. Lett..

[31] Siegman AE (1986). Lasers.

[32] Gigan S, Lopez L, Treps N, Maître A, Fabre C (2005). Image transmission through a stable paraxial cavity. Phys. Rev. A.

[33] Gardiner CW, Collett MJ (1985). Input and output in damped quantum systems: Quantum stochastic differential equations and the master equation. Phys. Rev. A.

[34] Kim MS, De Oliveira FAM, Knight PL (1989). Properties of squeezed number states and squeezed thermal states. Phys. Rev. A.

